# Lexical Feedback in the Time-Invariant String Kernel (TISK) Model of Spoken Word Recognition

**DOI:** 10.5334/joc.362

**Published:** 2024-04-26

**Authors:** James S. Magnuson, Heejo You, Thomas Hannagan

**Affiliations:** 1 BCBL: Basque Center on Cognition, Brain & Language, Donostia-San Sebastián, Spain; 2 Ikerbasque: Basque Foundation for Science, Bilbao, Spain; 3 Department of Psychological Sciences and CT Institute for the Brain and Cognitive Sciences, University of Connecticut, Storrs, CT, USA; 4 Hyundai Motor Group Robotics LAB, Uiwang, South Korea; 5 Stellantis Group, The Netherlands

**Keywords:** Computational models, neural networks, spoken word recognition, interaction, feedback

## Abstract

The Time-Invariant String Kernel (TISK) model of spoken word recognition (Hannagan, Magnuson & Grainger, 2013; You & Magnuson, 2018) is an interactive activation model with many similarities to TRACE (McClelland & Elman, 1986). However, by replacing most time-specific nodes in TRACE with time-invariant open-diphone nodes, TISK uses orders of magnitude fewer nodes and connections than TRACE. Although TISK performed remarkably similarly to TRACE in simulations reported by Hannagan et al., the original TISK implementation did not include lexical feedback, precluding simulation of top-down effects, and leaving open the possibility that adding feedback to TISK might fundamentally alter its performance. Here, we demonstrate that when lexical feedback is added to TISK, it gains the ability to simulate top-down effects without losing the ability to simulate the fundamental phenomena tested by Hannagan et al. Furthermore, with feedback, TISK demonstrates graceful degradation when noise is added to input, although parameters can be found that also promote (less) graceful degradation without feedback. We review arguments for and against feedback in cognitive architectures, and conclude that feedback provides a computationally efficient basis for robust constraint-based processing.

## 1. Introduction

Consider the speech signal. A series of rapid, overlapping articulatory events creates acoustic patterns that human listeners can map onto series of segments (consonants and vowels). Cues to word boundaries are rare and probabilistic; clear breaks in the signal are more likely to occur within words than between words in fluent speech (Cole, Jakimik, & Cooper, 1980; Lehiste, 1960). Even if listeners could perfectly extract a speaker’s intended segments from the speech signal in a bottom-up fashion (a virtual impossibility given phonetic and phonological processes such as coarticulation, assimilation, and reduction), considerable challenges would remain. Segment sequences must be mapped onto words in memory. Words are distinguished by order (the orderings of /k/, /æ/, and /t/ as /kæt/, /tæk/ and /ækt/ correspond to CAT, TACK, and ACT) and elements can be repeated (e.g., /to/ vs. /tot/, i.e., TOE vs. TOTE), so the encoding scheme for spoken word recognition must represent order and repeated elements. Recognition of *embedded words* must be avoided; when CATALOG is uttered, listeners hear the intended word, and are apparently unaware that they have also heard patterns that correspond to CAT, AT, A, CATTLE, LAW, and LOG (depending on dialect), or even a possible 3-word sequence (CAT A LOG). The system must tolerate variability that emerges from phonological processes such as assimilation that merge or alter phonetic properties of segments (e.g., GREEN BEAN may be realized as /grimbin/; e.g., Gow, 2003), and reductions that alter segments (e.g., TO as /tə/, or KIND OF as /kaində/) or even remove them. For example, RECOGNIZE SPEECH may reduce to /rɛk^naispit∫/. At first, one might consider that such an example could be disambiguated lexically, except that a plausible alternative parse would be WRECK A NICE BEACH (Picone, Goudie-Marshall, Doddington, & Fisher, 1986). In such cases, a broader semantic context might be needed to constrain lexical mapping and arrive at the correct parse.

In grappling with these challenges, theories of spoken word recognition have come to agree on three fundamental principles: *As* a word is heard (*incrementality*), words are activated (or their probability is estimated; Norris & McQueen, 2008) based on degree of phonetic overlap with the input and their prior probability (*probabilistic similarity mapping*), and activated words compete for recognition (*parallel competition)*.^1^ Theories differ in similarity metrics, and in the mechanisms they posit for achieving parallel activation and implementing and resolving competition (ranging from lateral inhibition to bottom-up or top-down inhibition, or competition implicit in Bayesian normalization; for a review, see Magnuson, Mirman & Harris, 2012). However, a particularly vexing problem is how to deal with the sequential nature of speech, as we discuss next.

### 1.1. The problem of sequence encoding

**Figure 1:**
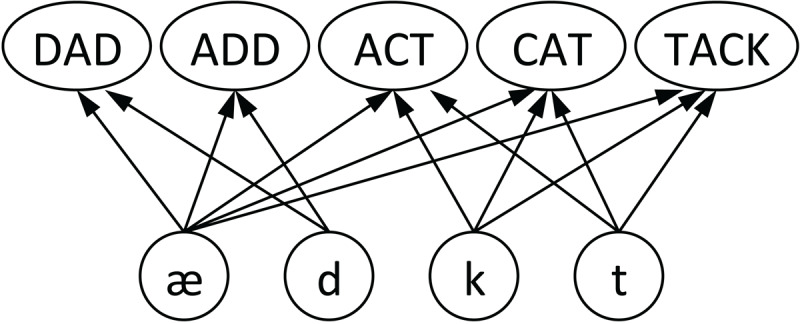
A simple word recognition network incapable of encoding temporal order or repeated phonemes (Magnuson, 2018a).

Sequence encoding is a fundamental challenge for models of spoken word recognition; speech unfolds over time, and representing phonological word forms entails representing temporal order (CAT vs. TACK, i.e., /kæt/ vs. /tæk/) and repeated elements (SOUL vs. SOLO, i.e., /sol/ vs. /solo/). To illustrate this challenge, consider the simple network in Figure 1. Here, the only connections are forward ones from phoneme nodes to word nodes. Note that such a network cannot encode temporal order. Any word node receiving input from /k/, /æ/, and /t/ in any order (i.e., ACT /ækt/, CAT /kæt/, TACK /tæk/, or nonwords /tkæ/, /ktæ/, or /ætk/) would be equally activated by any ordering of the three phonemes. Neither could such a network distinguish words with the same constituent phonemes but differing in repeated elements (SOUL vs. SOLO). The second /o/ in /solo/ would simply be more evidence that /o/ had occurred; the network cannot represent two instances of /o/ in different temporal positions.

Note that a model like this could be used to investigate many aspects of word recognition. In fact, the Merge model (Norris et al., 2000) has this structure (as well as lateral inhibition), and can simulate many important aspects of spoken word recognition, despite being unable to encode order or repeated elements. Avoiding these challenges can only be a temporary simplifying assumption, however. Ultimately, models of spoken word recognition must grapple with the representation of order and repeated elements.

The TRACE model (McClelland & Elman, 1986) takes an innovative approach to the problem. TRACE translates time to space, by creating time-specific duplicates of feature, phoneme, and word nodes. A template for CAT is maximally activated by strongly activated /k/, /æ/,^2^ and /t/ phonemes aligned with a word node standing for CAT.

**Figure 2:**
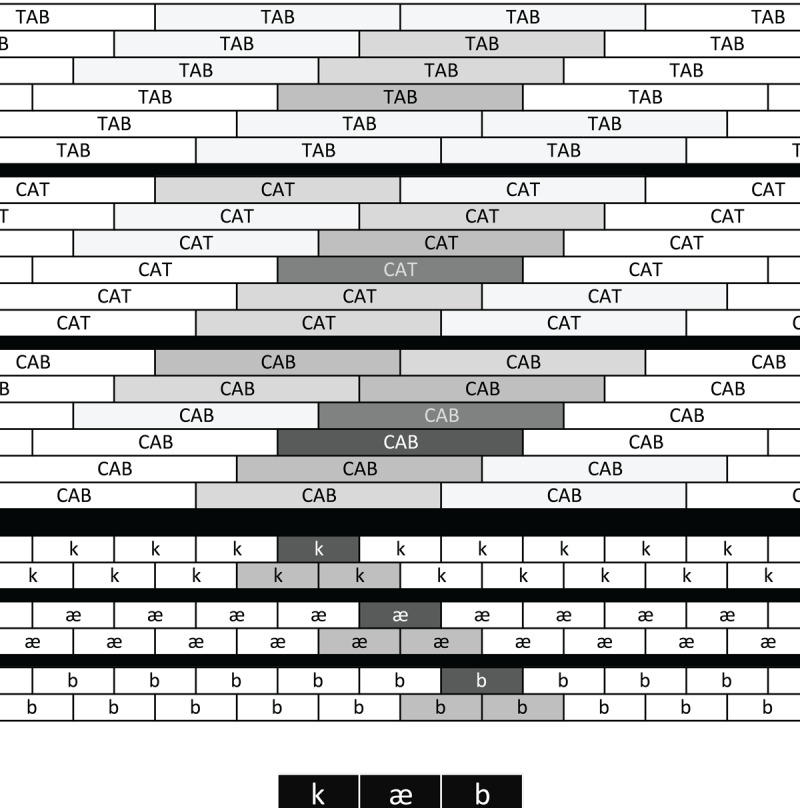
TRACE’s time-as-space encoding (Magnuson, 2018b). At the bottom, inputs corresponding to /k/, /æ/, and /t/ have specific alignments (in TRACE, these would be distributed representations of over-time pseudo-spectral features). Those inputs activate phoneme templates aligned with them, which in turn activate aligned words. Darkness of shading indicates degree of activation. The maximally-activated copies of CAB, CAT and TAB are those aligned with the input, though degree of activation reflects amount and temporal distribution of phonetic overlap (CAB > CAT > TAB).

Figure 2 contains a schematic outlining the complex relationships between feature, phoneme, and word nodes in TRACE. At the bottom of the figure, black cells stand in for the distributed vector of pseudo-spectral representations used as TRACE inputs. Their horizontal extent represents their temporal extent. Although feature patterns for adjacent phonemes overlap in TRACE (providing a coarse analog to coarticulation), for the sake of simplicity, we do not attempt to depict that overlap here.

At each time step *t* in a TRACE simulation, pseudo-spectral input patterns are applied. Feature nodes aligned with input slice *t* (that is, time-specific feature nodes) are activated by the bottom-up input at time *t*. Subsequently (from step *t + 1* onward), bottom-up input is not applied at slice *t*. However, feature detectors aligned at slice *t* that were activated by input continue to be active for many time steps, because their activations are a summative combination of their bottom-up input and previous activation. The latter is scaled by a *decay* parameter, such that a unit’s activation will eventually diminish to a defined baseline level in the absence of new input. Similarly, phoneme nodes are aligned at specific time slices, and receive input from feature nodes aligned with them in time. As long as the aligned feature nodes are active, the aligned phoneme nodes will receive bottom-up input. Phoneme nodes’ activations are a function of bottom-up input and decay-scaled prior activation, as well as lateral inhibition from other phoneme nodes with which they overlap in time, and lexical feedback (described below). Phoneme nodes send bottom-up activation to nodes corresponding to words containing them *that are aligned (at least partially) in time with the phoneme node*. Word nodes also send feedback to phoneme nodes that send them bottom-up input. As “time” progresses in a TRACE simulation, inputs aligned with specific time points activate aligned features, phonemes, and words. This time-specific “reduplication” strategy – aligning copies of each feature, phoneme, and word in memory with specific time points – allows TRACE to represent temporally ordered sequences, including sequences with repeated elements. Thus, given the input /dæd/ (DAD), the first and second instances of /d/ would activate independent /d/ nodes.

This reduplication strategy is frequently criticized. Indeed, McClelland and Elman (1986) discussed plausibility concerns (p. 77). Some have argued that this scheme is simply implausible (e.g., Grossberg & Kazerounian, 2011; Norris, 1994), largely because of the numbers of nodes and connections it would take to implement a realistic phoneme inventory and lexicon. Magnuson (2015) presents a case for the TRACE architecture as a kind of echoic memory. Hannagan et al. (2013) estimate how many nodes and connections a realistically-sized version of TRACE would require, and estimate that a version with 40 phonemes and 20,000 words would require ~1.3 million nodes and more than 40 billion connections. Given estimates that the human brain contains approximately 86 billion neurons and 150 trillion synapses (Azevedo et al., 2009), it is not clear that we can rule out the TRACE solution based on intuitions about the plausibility of numbers of units and connections required. However, it does raise the question of whether a more compact representation might be possible, which leads us to a discussion of the TISK model.

### 1.2. Origins and innovations of TISK

The idea of TISK originally came from discussions between Jonathan Grainger and TH, and eventually included JM. The aim was to keep the explanatory power of the TRACE model while dispensing with its duplicated time-specific units. Hannagan et al. (2013), inspired by models of visual word recognition developed by Grainger and others using *open bigram codes* (Whitney, 2001; Grainger & van Heuven, 2003; Dehaene et al., 2005), asked whether a simpler interactive activation model of spoken word recognition could be implemented with a variant of *open diphone coding*. Open diphones are adjacent or non-adjacent phoneme pairs that occur in a string. For example, the (ordered) open diphones of ACT (/ækt/) are /æk/, /kt/, and /æt/ (see Table 1 for several more examples). As it turns out, such lists are highly distinctive. To encode the lists in a length-independent fashion, we can create a phoneme × phoneme matrix (corresponding to all possible diphones),^3^ and simply enter the count of each diphone for a word. This then is a kind of *string kernel*^4^ for words: we can manipulate or compare representations of words of any size through vector/matrix operations (i.e., the operations are identical since they are computed over matrices).

**Table 1:** Examples of ordered open diphones.


WORD	ORDERED OPEN DIPHONES

CAT	kæ, kt, æt

TACK	tæ, tk, æk

ACT	æk, æt, kt

DAD	dæ, dd, æd

ADD	æd

SOUL	so, sl, ol

SOLO	so x 2, sl, ol, oo


**Figure 3:**
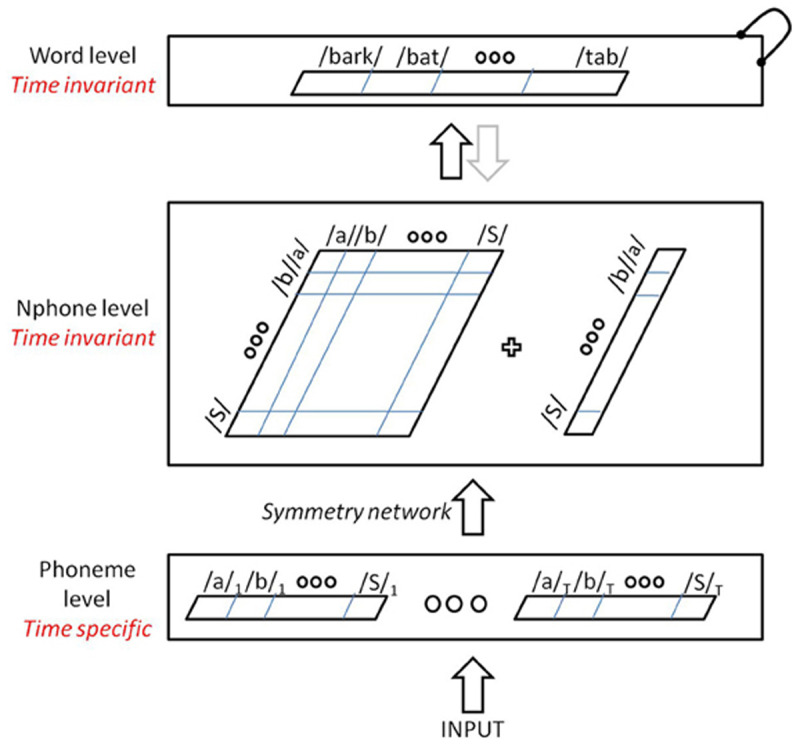
Overall TISK architecture (Figure 3 from Hannagan et al., 2013). Inputs are presented one at a time on time-specific copies of each possible phoneme. Phonemes activate corresponding diphones and single nodes in the N-phone layer. N-phone units activate corresponding words. Lateral inhibition governs lexical competition (indicated by knobbed recurrent link in top right). The greyed out arrow from words to N-phones indicated that the original TISK model did not have lexical feedback (which is the only structural alteration in the model introduced in this paper). The symmetry network (not shown; see Figure 4 from Hannagan et al., 2013) allows an input like /ba/ to activate both the /ba/ and /ab/ diphones, but activates the diphone corresponding to the input order much more strongly. See Hannagan et al. (2013, pp. 5–6) for details.

TISK’s architecture is presented schematically in Figure 3. Time-specific phoneme input nodes feed to time-invariant N-phone nodes (corresponding single phone and diphone nodes), but via what Hannagan et al. dubbed a *symmetry network* (in recognition of prior work on the topic by Shawe-Taylor, 1993). The symmetry network does not activate all open diphones equally. It privileges ordered diphones and activation is inversely proportional to distance between diphone members (e.g., /st/ would be less activated by SPOT than STOP). This followed work by Dandurand, Hannagan and Grainger (2013) showing that weight gradients can emerge in models of visual word recognition trained to be invariant to the location of the word input on a simulated retina. It also built on work by Hannagan and Grainger (2012), who noticed the similarity between N-gram schemes for visual word recognition, and a versatile technique called “string kernels” that has been used in text classification (Lodhi et al. 2002) and computational biology (Leslie & Kuang, 2004). Building on these two strands of work, the TISK symmetry network uses weight gradients as well as gating connections to accurately activate N-phone nodes, even in the presence of repeated phonemes. For more details about the symmetry network and TISK more generally, see Hannagan et al. (2013). Note that the full code for TISK is freely available (You & Magnuson, 2018; https://github.com/maglab-uconn/TISK1.0; also, updated code from this project is also available at https://github.com/maglab-uconn/TISK_FEEDBACK).

TISK thus may be viewed as a potential successor to or extension of TRACE that addresses the critique of time-specific nodes. However, TISK has not been tested on the entire broad range of results that TRACE accounts for (Magnuson & Crinnion, 2022). Hannagan et al. (2013) focused on a subset of particularly critical phenomena in spoken word recognition (the time course of phonological competition, and the relations between a variety of lexical dimensions and recognition time in TISK vs. TRACE) to establish initial plausibility of the model. However, they did not consider a broad class of phenomena in spoken word recognition that have particular relevance for ongoing theoretical debates: apparent *top-down lexical effects*. Our primary goal here is to address this gap.

### 1.3. Feedback and theories of spoken word recognition

A particularly salient point of disagreement in theories of spoken word recognition concerns top-down feedback from words to sublexical representations. TRACE (McClelland & Elman, 1986) is an interactive-activation model with arguably the deepest and broadest coverage of spoken word recognition (cf. Magnuson et al., 2012, Magnuson & Crinnion, 2021). Top-down effects in TRACE emerge from lexical-phonemic feedback. In contrast, Norris, McQueen, and Cutler (2000; see also 2016) have argued that purely feedforward systems can do anything a feedback system can do, so long as they include a mechanism for post-perceptual behavior consistent with top-down influences (e.g., via post-lexical integration of phonemic input and lexical knowledge). As TISK is a derivative of TRACE, our goal here is to make TISK more comparable to TRACE and assess the possibility of adding feedback to TISK. Without feedback, top-down effects are out of scope for TISK. We will return briefly to theoretical disagreements concerning feedback in the Discussion.

Consider two important top-down effects in spoken word recognition. First, there is the Ganong (1980) effect, where phoneme identification is influenced by lexical status. For example, compared to a nonword continuum between *iss* and *ish*, where participants are asked to identify the final consonant, identification shifts towards /s/ if the continuum is instead between a word and nonword pair like kiss-*kish, but towards /∫/ given *fiss-fish. Thus, either lexical context modulates phonetic perception (the interactive or feedback assumption), or it has a post-perceptual influence on responses (the feedforward assumption). Another fundamental top-down effect in spoken word recognition is phoneme restoration (Samuel, 1981a, 1981b, 1996, 1997; Warren, 1970). If a phoneme in a word is replaced by silence, it leaves a salient gap, and participants have no trouble reporting that the word is not intact and can identify which phoneme is missing. In contrast, when a phoneme is replaced by noise, participants typically report that the word is intact but has noise added to it. They have difficulty specifying which phoneme the noise is aligned with, and report perception consistent with lexical context (e.g., if noise, denoted as #, replaces a phoneme in the word *after*, the noise is heard as /t/ in /æf#^r/ but as /f/ in /æ#t^r/). This implies that noise provides enough bottom-up support for the missing phoneme to be filled in, either perceptually via lexical feedback or via post-perceptual lexical integration.

While such top-down effects are quite salient, a less obvious benefit of feedback is to make models more robust to noise. Top-down feedback (in concert with lateral inhibition in TRACE; Magnuson et al., 2024) promotes accuracy and faster processing given noisy inputs (Magnuson et al., 2018). While more subtle, this may be the more important impact of feedback.

## 2. Adding lexical feedback to TISK

Again, there are several reasons to add feedback to TISK. Any comprehensive model of spoken word recognition must be able to account for top-down effects, and feedback allows TRACE to plausibly simulate many such effects (McClelland & Elman, 1986). As discussed above, however, at least some effects considered to be “top-down” can be simulated without feedback (Norris et al., 2000). However, graceful degradation is another important motivation for feedback in interactive activation models (Dell, Chang & Griffin, 1999; McClelland & Elman, 1986 [e.g., pp. 6–7]; McClelland & Rumelhart, 1981, 1989), which turns out to have important implications for the feedback vs. autonomy debate. Graceful degradation seems to be less familiar to most cognitive scientists (e.g., it received no discussion in the Norris et al., 2000, target article or in the accompanying commentaries), although it is one of the original, primary motivations for feedback in interactive activation models (for example, when noise is added to inputs, feedback promotes gradual declines in performance rather than an abrupt collapse; McClelland & Rumelhart, 1981).

These points direct us to a clear agenda for simulations with feedback (from words to N-phones) added to TISK. First, can we identify a non-zero feedback parameter that will (a) afford plausible top-down effects while allowing robust word recognition, without impeding the model’s ability to simulate the phenomena attested by Hannagan et al. (2013), including (b) the time course of phonological competition and (c) item-specific correlations with TRACE and (d) lexical dimensions (word length, numbers of different competitor types, etc.)? Finally, (e) will feedback in TISK allow the model to exhibit graceful degradation given noisy inputs (i.e., will feedback preserve accuracy and processing efficiency)? We address these issues in the following order: parameter discovery, replication of earlier simulations (time course, similar item-specific recognition times as for the original TISK model and TRACE, similar item-specific correlations with lexical dimensions), simulations of crucial top-down phenomena in spoken word recognition, and performance in noise (testing for graceful degradation). All code required to reproduce our simulations, analyses, and figures is available at https://github.com/maglab-uconn/TISK_FEEDBACK.

### 2.1. Simulation 1: Time course and Lexical Dimensions

#### 2.1.1. Parameters

We used a trial-and-error process for parameter exploration. We began with a value of positive feedback from words to their constituent N-phones. We assessed mean accuracy over the 211-word (original TRACE) lexicon, and if accuracy was lower than approximately 80%, we examined errors for clues as to what was impeding accuracy. If we found a parameter setting that would allow reasonable accuracy, we then examined the model’s ability to simulate top-down effects (with phenomena like those discussed below). If feedback was not strong enough for plausible top-down effects, or if error patterns implied parameter changes were needed, we would adjust parameters and retest. We iterated this process, gradually increasing our accuracy threshold.

After a few iterations, we determined that there were three key parameters that could be adjusted to provide the full complement of desired outcomes (a–e above). First, of course, we needed positive feedback from words to constituent N-phones. Second, feedback tended to cause resonance between word and N-phone layers that would lead to the activation of too many words. For example, given the input /dal/ (DOLL), the lexical node for DOLL would send feedback to /d/, /a/, /l/, /da/, /dl/, and /al/ nodes at the N-phone level. These would enhance activation of *doll*, but also any word containing any of these elements (e.g., SADDLE and DRILL would contain /dl/), allowing them to send feedback to elements that had not occurred. We discovered that we could avoid “runaway” activation by both increasing decay at the N-phone level and by including a small amount of negative feedback to a word’s non-constituents (i.e., a small amount of inhibition to every N-phone or single phone that is not part of a word, similar to top-down inhibition in early interactive activation models, e.g., McClelland & Rumelhart, 1981). Table 2 lists key parameters we considered altering, with the three parameters that were ultimately altered in bold font. We have not searched the parameter space exhaustively. However, our explorations suggest that stable performance requires ratios among parameters similar to those in Table 2.

**Table 2:** Original (without feedback) parameters for TISK, and parameters that promote high performance with feedback. Parameters in the ‘optimized without feedback’ column that differ from original parameters are in bold. Parameters in the ‘optimized with feedback’ column that differ from parameters in the ‘optimized without feedback’ and/or ‘original TISK’ columns are also in bold.


PARAMETER	ORIGINAL TISK	OPTIMIZED WITHOUT FEEDBACK	OPTIMIZED WITH FEEDBACK

Input phoneme decay	0.010	0.001	0.001

N-phone decay	0.001	0.001	**0.100**

Word decay	0.010	**0.050**	0.050

Phoneme to N-phone	1.000	**0.100**	0.100

Diphone to word	0.050	0.050	0.050

Single phone to word	0.010	0.010	0.010

Word to word inhibition	–0.005	–0.005	**–0.010**

Positive word to N-phone feedback			**0.150**

Negative word to N-phone feedback			**–0.050**


We also considered that the original TISK parameters might not provide the best possible performance in noise without feedback. We therefore explored the parameter space without feedback with the aim of finding parameters that would allow the model to continue to exhibit fundamental target behaviors described below while maximizing performance in noise. We present details of our parameter space exploration for models with and without feedback in Appendix 1. For now, because the most robust parameters for the model without feedback differ from the original TISK parameters, we will present results in the following simulations using the new parameter set (while noting that the original TISK model and the version with feedback and parameters optimized for graceful degradation differ only slightly and qualitatively in the following simulations – with the exception, of course, of the final graceful degradation simulations).

**Figure 4:**
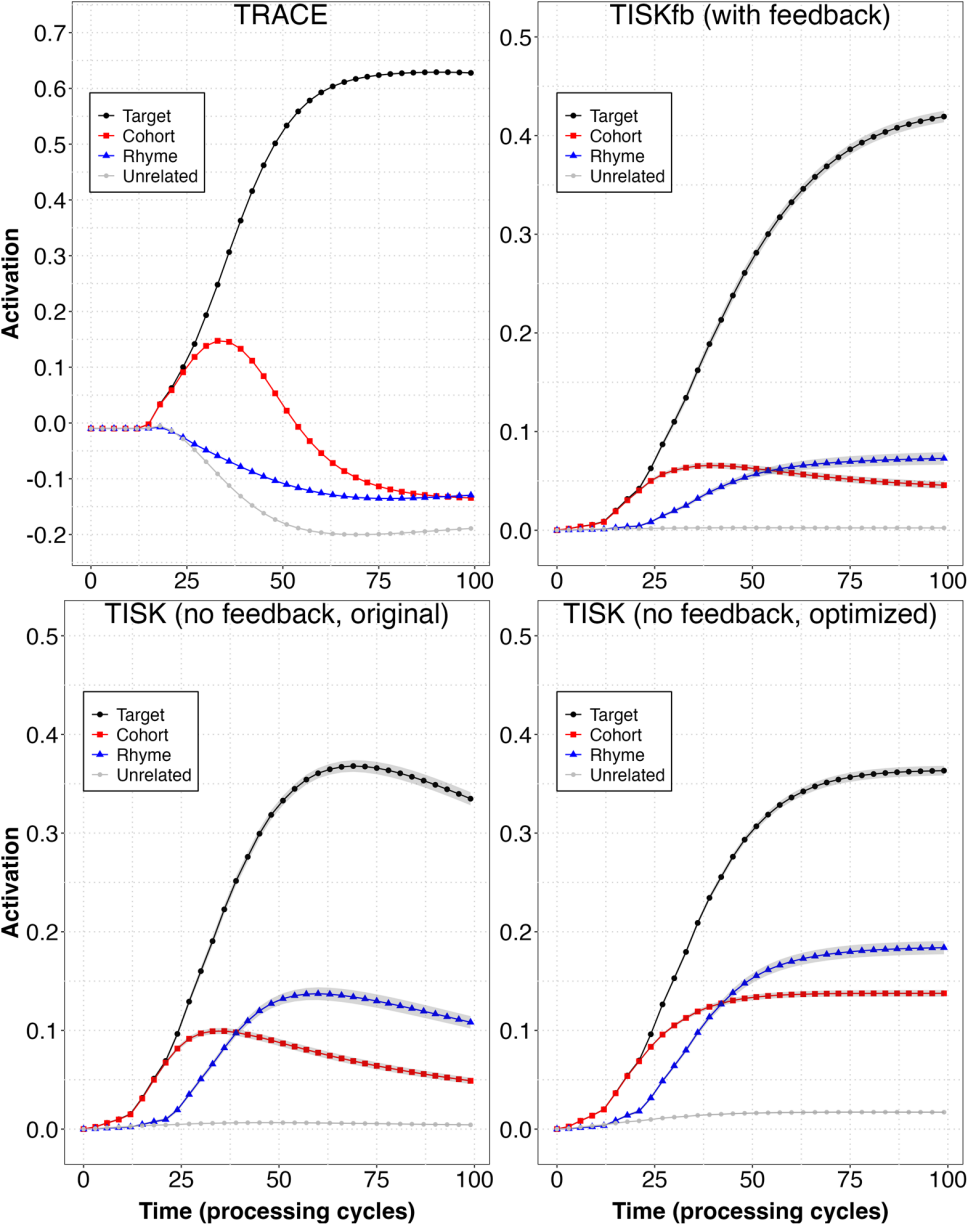
Mean time course for targets and different classes of competitors in TRACE and TISK with and without feedback (including the original model, as well as the version with parameters ‘optimized’ for graceful degradation, as detailed later). Each line represents the mean for a class of items over all 211 words in the original TRACE lexicon. *Cohorts* overlap in the first two phonemes. *Rhymes* overlap in all but the first phoneme. *Unrelated* is the mean activation of all words in the lexicon. Ribbons indicate standard error.

Before turning to top-down effects, let us consider whether TISK performs similarly with and without feedback on the tasks evaluated by Hannagan et al. (2013). Figure 4 addresses this by first examining mean activation over time for different *categories* of potential phonological relatives. To conduct this comparison, we conducted 211 simulations with TRACE and with two versions (with and without feedback) of TISK. For each model, there were 211 simulations (one for each word in the original TRACE lexicon). For every target word, we tracked target activation over time, as well as the mean activation of every item in two categories of phonological relatives (cohorts and rhymes) over time (e.g., for /dal/, the activation of every word beginning /da/ would be included in the [onset] cohort mean, and every three-phoneme word ending in /al/ would be in the rhyme category). If a word had no relatives in a category, it would not contribute to the mean for that category. As a baseline reference, we simply tracked the mean activation of *all* words; given 211 words, this mean approaches the minimum possible activation value. Although the mean values are somewhat damped when feedback is added to TISK, the crucial consideration is that the rank ordering of competitors is similar for all three models.^5^

**Figure 5:**
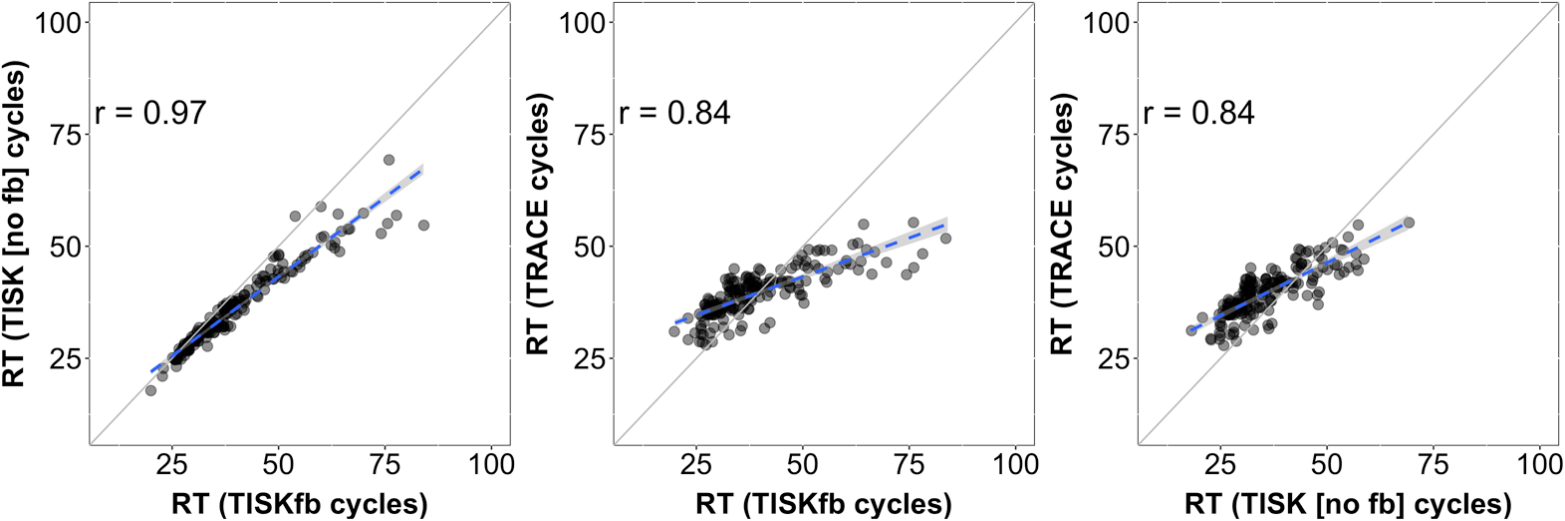
RT correlations for original TISK (without feedback), TISKfb (TISK with feedback), and TRACE. Left panel: TISKfb vs. TISK. Middle panel: TISKfb vs. TRACE. Right panel: original TISK vs. TRACE. Diagonal grey lines indicate the identity line, dashed lines indicate best linear fit.

Figure 5 extends our examination of how similar the performance of TISK is (with and without feedback) to TRACE by comparing item-specific recognition times (RTs) for each model. Recognition time was operationalized as the cycle at which the target word exceeded all other word’s activations by at least 0.05 and then continued to exceed all others by that amount for at least 10 cycles (cf. Hannagan et al., 2013), and subsequently remained the most activated word until the end of the simulation. Mean accuracies were 100% for TRACE, 99% for the original TISK without feedback (TISK), and 97% for TISK with feedback (TISKfb). As can be seen in Figure 5, item-specific RTs for correctly recognized items were remarkably similar for the three models.

**Figure 6:**
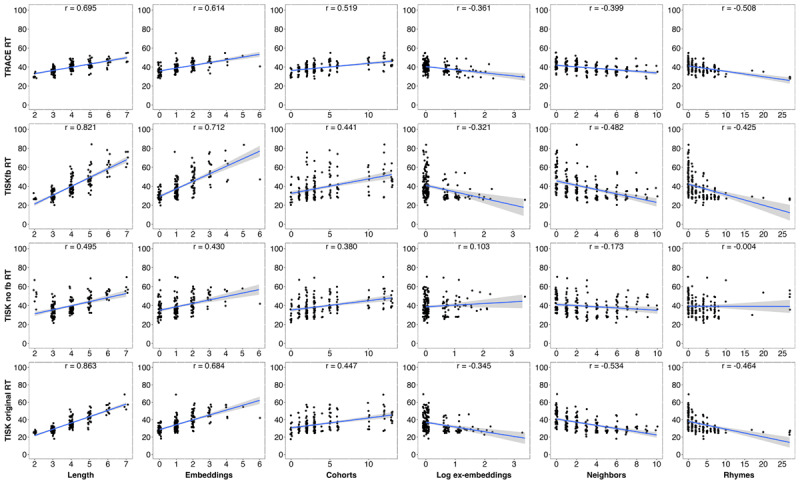
item-specific RTs in TRACE, TISKfb (with feedback), TISK without feedback with parameters optimized for noise, and original TISK (without feedback), as a function of lexical dimensions for the 211-word TRACE lexicon. Dimensions: *Length* is number of phonemes, *Embeddings* is how many words embed within the target word (e.g., CAB and IN embed in CABINET), *Onset competitors are cohorts* (words overlapping in the first two phonemes), *ex-Embeddings* are the number of words the target word embeds into (e.g., CAB embeds in CABINET, CABARET, etc.), *Neighbors* are the number of words differing from the target by no more than a 1-phoneme deletion, addition, or substitution (so-called DAS neighbors), and *Rhymes* items are items that mismatch the target only at the first phoneme (by deletion, addition, or substitution; e.g., for CAT, these would include AT, SCAT, and BAT).

Figure 6 goes deeper by examining how item-specific RTs in the three models (plus a fourth variant: TISK without feedback with parameters optimized for accuracy in noise, as described in Simulation 5) relate to several lexical dimensions: word length (in phonemes), number of embeddings (words embedded in the target, e.g., CAT has AT embedded within it), number of cohort (onset) competitors (overlapping in the first two phonemes), number of “ex-embeddings” (words the target embeds within, e.g., CAT embeds within CATALOG), number of “DAS” neighbors (i.e., words differing from the target by a single phonemic deletion, addition, or substitution; Luce & Pisoni, 1998), and number of “rhyme” items (words differing from the target only in first position, whether by deletion, addition, or substitution; e.g., CAT’s rhymes include SCAT, BAT, MAT, SAT, and AT). The dimensions are ordered according to the sign and magnitude of their prediction on RT; longer words are recognized more slowly, having more embeddings or cohorts is associated with slower RT, and having more ex-embeddings, neighbors or rhymes is associated with faster RT. The potential reasons for these relationships is beyond the scope of this paper (Magnuson, in preparation, discusses this in detail); our focus is instead the similarities between models. All models show the similar patterns, and are even generally similar in the strength of each correlation (although TISK without feedback with parameters optimized for performance in noise [third row] differs for ex-embeddings, neighbors and rhymes, as we discuss in Section 2.5).

The results from Simulation 1 demonstrate that we can add feedback to TISK without disrupting the model’s similarity to TRACE. The time course of different kinds of phonological competition are quite similar, and TISK retains its high similarity to TRACE in item-specific RTs with feedback on, and there are only very subtle quantitative differences in item-specific RTs between TISK with and without feedback apparent in our examination of how a variety of lexical dimensions relate to recognition time. With this fundamental consideration of prior results resolved, we can turn to the details of specific top-down effects.

### 2.2. Simulation 2: Ganong effect

For Simulation 2, we compared the ability of TISK with and without feedback to simulate the *Ganong effect* (Ganong, 1980). In the Ganong paradigm, we begin with a continuum from one phoneme to another (e.g., changing gradually from /s/ to /∫/, e.g., *ess* to *esh*) and establish a baseline identification pattern across the continuum (e.g., rate of “s” [vs. “sh”] responses at each step). If we add context such that the continuum changes from a word to a nonword (e.g., from *bus* /b^s/ to *buhsh /b^∫/, or from *russ /r^s/ to *rush* /r^∫/), human listeners’ identification rates will change. Specifically, they will make more responses consistent with the lexical endpoint, typically shifting the category boundary away from the lexical endpoint (e.g., for /b^s to /b^∫/, they will make more “s” responses, and the shift to “sh” responses will happen closer to the unambiguous /∫/ endpoint).

**Figure 7:**
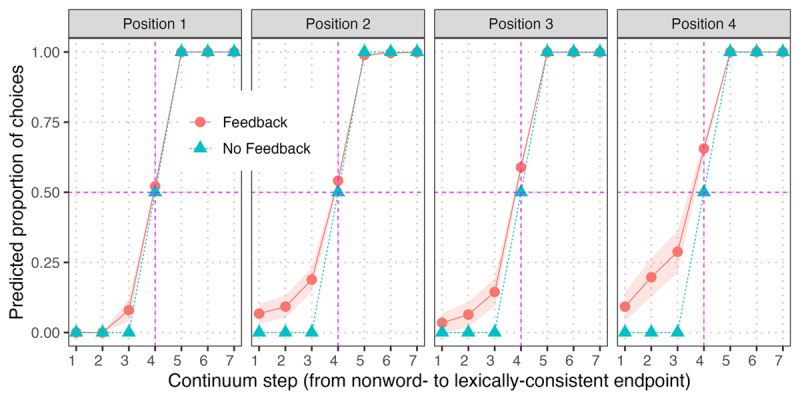
Lexical effects on phoneme activations (Ganong effects) for ten 4-phoneme words (Simulation 2). We observe robust Ganong effects (lexical restoration) at each position with lexical feedback enabled, with stronger effects in later positions. The key results are that (a) greater ambiguity is apparent for continuum steps near the nonword endpoint and (b) the upward shift for the center continuum step (4). Error ribbons indicate standard error.

To simulate the Ganong effect with TISK, we selected ten 4-phoneme words from the lexicon (*appeal, box, boost, duty, greet, least, school, shield, screw*, and *ugly*). For each word, we conducted a Ganong simulation at each position by creating nonwords replacing the phoneme at the critical position, and then creating a continuum blending from the original phoneme to the replacement. For example, for /duti/ (duty), we created continua between /duti/ and four nonwords: /buti/, /d^ti/, /duri/, and /dut^/. So in Figure 7, for the *Position 4* panel, the relevant simulation for /duti/ would be the /duti/ to /dut^/ continuum. We aggregated results by averaging activations of the lexically-consistent phoneme and its nonword replacement, and calculating ‘predicted proportion of choices’ as the ratio of the peak activations for those two phonemes. In Figure 7, we observe robust Ganong effects (lexical restoration) at each position in the model with feedback, with stronger effects in later positions (consistent with TRACE simulations of phoneme restoration in TRACE reported by Magnuson, 2015). This increase of the effect at later positions is a result of greater lexical activation as more bottom-up input is received. Thus, feedback allows TISK to simulate the Ganong (lexically-driven phoneme restoration) effect.

### 2.3. Simulation 3: Retroactive effects of feedback

In Simulation 3, we focus on retroactive influences of lexical feedback on the activation of phonemes given ambiguous input, where the disambiguating lexical context only emerged at the final phoneme (so-called *right-context effects*; see simulations described by McClelland & Elman [1986] on the following pages for related results: pp. 27, 29, 30 [their Figures 8, 11]; pp. 66–69). For this simulation, we used the lexical items *plug* and *blush*. If we replace the onsets of these items with a stimulus halfway between /p/ and /b/ (denoted by /#/), we create an ambiguity that will be sustained until the final phoneme is presented. We conducted simulations where the inputs were either the clear lexical inputs /pl^g/ or /bl^∫/ to establish baseline activations for /p/ and /b/ (we added *blush* to the TRACE lexicon for this simulation; note also that *plush* was not in the lexicon). Then we conducted simulations where the input was /#l^g/ (disambiguated as *plug* at the final phoneme) or /#l^∫/ (disambiguated as *blush* at the final phoneme).

**Figure 8:**
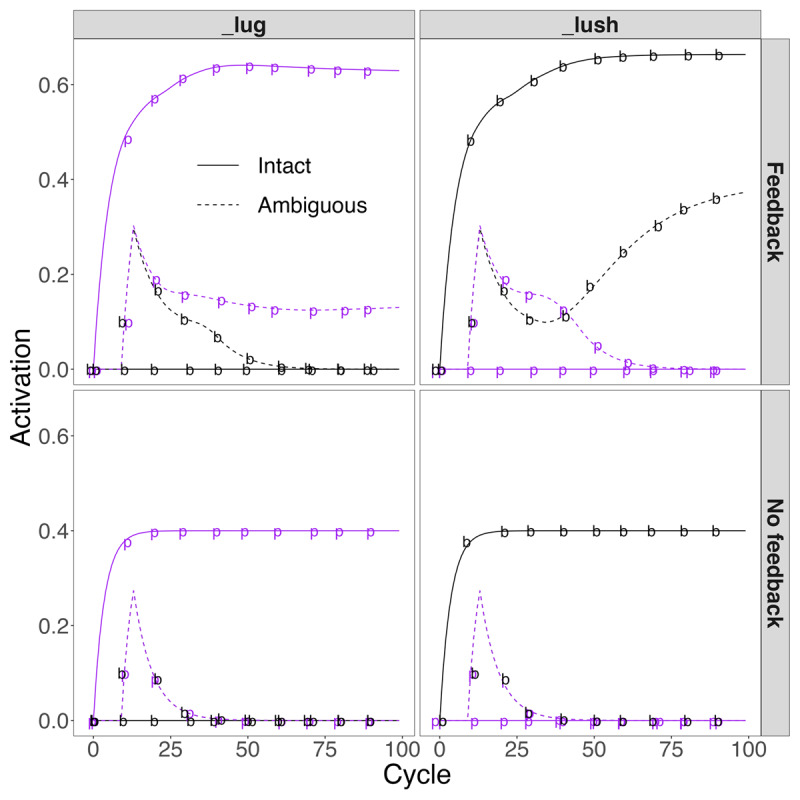
Retroactive phoneme restoration by following context (Simulation 3). In the lexicon, *plug* and *blush* are words, but *blug and *plush are not (even though *plush* is a word in English). Note that the delayed activations of ambiguous phonemes is due to failure to reach the activation threshold from the initial input. The discrete delay of 10 cycles is due to new TISK inputs ‘arriving’ every 10 cycles.

The results are plotted in Figure 8. Left panels show results with the _lug context (either /pl^g/ when it is intact, or /#l^g/ when it is ambiguous); right panels show results with the _lush context (/bl^∫/ when it is intact, or /#l^∫/ when it is ambiguous). Upper panels show results with feedback; lower panels show results without feedback. In each panel, we plot activations for /p/ and /b/ given intact vs. ambiguous inputs. Without feedback (lower panels), lexical contexts have no effects, and the ambiguous stimuli drive equivalent activations of /p/ and /b/. With feedback (upper panels), the initial phase of activation is identical for both contexts because it is driven purely by the bottom-up input. As more context arrives, we see changes primarily in diminished decay of lexically-consistent phonemes (e.g., ambiguous /p/ in the upper left panel). However, the effects are different for the two ambiguous contexts, with differences emerging around cycle 20. The initial differences are stronger activation of /p/ than /b/ prior to disambiguation. This occurs because there are more words that begin with /p/ than /b/ in the model’s lexicon. The effects are stronger for the _lush context; this emerges because there are 4 items with the diphone /^S/ in the lexicon, but 7 with /^g/. Since the items activated by feedback will compete with the ambiguous onset position, having a smaller number of items sharing the pattern leads to greater ultimate activation. Thus, Simulation 3 shows clear retroactive effects of feedback.

### 2.4. Simulation 4: Phoneme restoration

In Simulation 4, we turn to another classic top-down effect using an analog to the *phoneme restoration* paradigm (Samuel, 1981a,b, 1996, 1997; Warren, 1970). In a phoneme restoration paradigm, a phoneme is replaced either with noise or with silence (typically in a lexical context where there is only one possible completion for the replaced phoneme, e.g., #*uxury* or *_uxury* [where # indicates noise and _ indicates silence] can only be restored as *luxury*). The two kinds of replacement yield very different effects. If a phoneme is replaced by noise, a listener typically reports hearing all the phonemes in the word, and will likely have difficulty identifying the precise location of the noise. If a phoneme is replaced by silence, the gap is salient, and listeners can report the precise location of the silence and which specific phoneme is missing. Another difference is that noise-replaced phonemes can drive *selective adaptation* (Samuel, 1997), as though the actual phoneme had been repeated, but silence cannot. The interpretation of this pattern is that noise provides sufficient bottom-up activation that the missing phoneme is “filled in” by feedback. As a result, the listener not only cannot reliably report which phoneme has been replaced, but is uncertain of the position of the noise. This means that the critical pattern a model must be able to simulate is (a) robust activation of a lexically-consistent phoneme when it is replaced with noise, but (b) weak or absent activation when it is replaced with silence (see Grossberg & Kazerounian, 2011, 2016 and Magnuson, 2015, for a debate about how phoneme restoration should be modeled).

To test TISK’s ability to simulate phoneme restoration with and without feedback, we used the same ten 4-phoneme words from Simulation 2. For each item, we conducted 48 simulations; 2 models (feedback of no feedback) × 4 phoneme positions × 6 input types (intact phoneme, silence replacement [the phoneme replaced by zero input], or noise replacement [silence plus noise with standard deviation of 0.2, 0.3, 0.4, or 0.8]). We examined the activations of the “expected” phonemes each position (e.g., /d/, /u/, /t/, and /i/ for duty) when they were intact versus when they were replaced with silence or increasingly strong noise. Again, in a successful simulation, replaced phonemes should be robustly activated given sufficient noise input, but should be activated weakly or not at all given replacement with silence.

**Figure 9:**
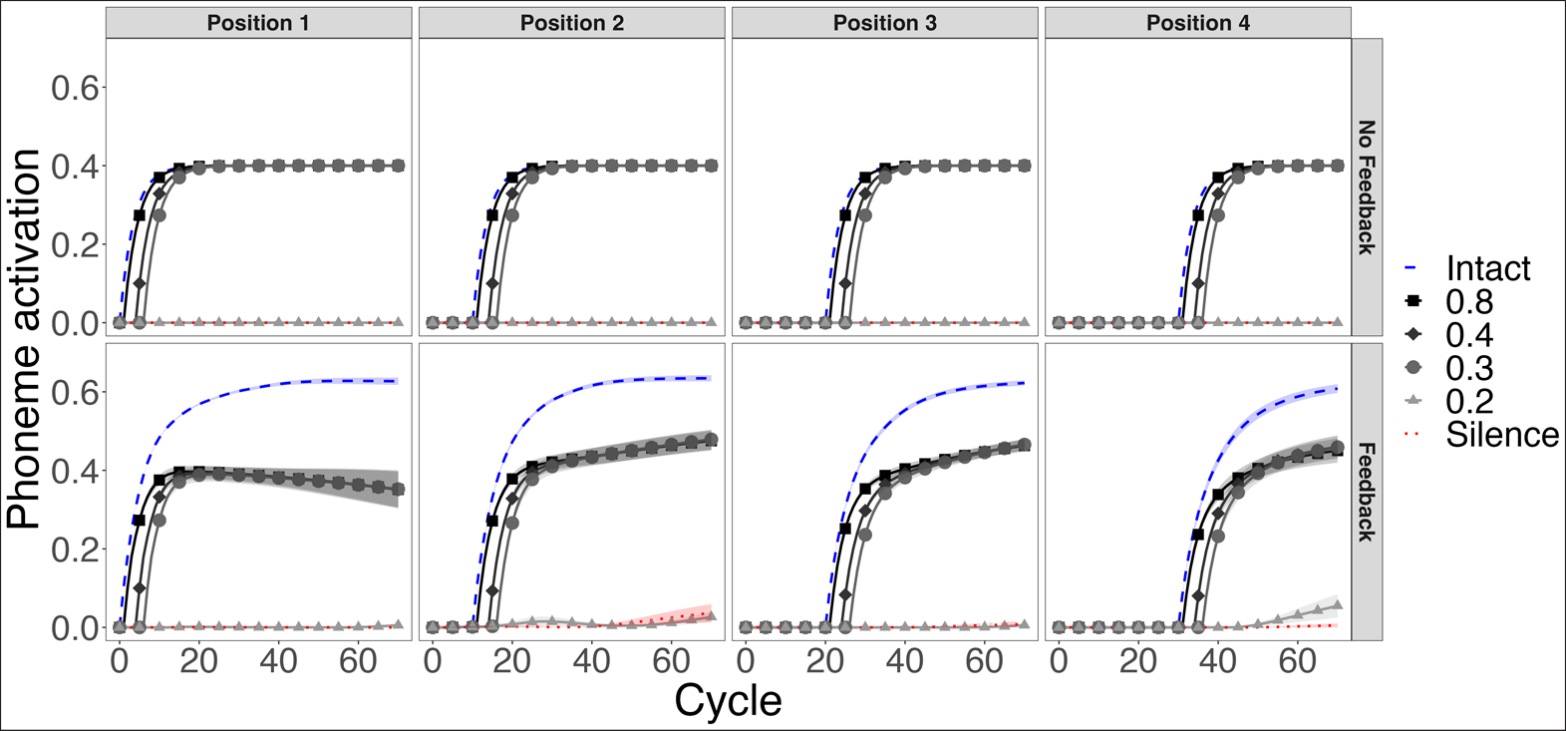
Phoneme restoration given noise vs. silence (Simulation 4). Mean results from simulations with ten 4-phoneme words. Top row: TISK without feedback. Bottom row: TISK with feedback. With feedback, moderate levels of noise (standard deviation ≥ 0.3) drive restoration, although the resulting activation is always less than that observed with the intact phoneme. Without feedback, noise level matters little, and even modest levels of noise drive expected phonemes to saturation. Note that phoneme activations remain at approximately 0 given silence replacement. Error ribbons depict standard error.

The results are shown in Figure 9. First, consider the results without feedback (top row). There is no activation whatsoever of the replaced phoneme given silence replacement or noise with standard deviation of 0.2, and slightly graded activations given noise, very near the level of activation observed for intact phonemes.^6^ In contrast, large lexical effects are readily apparent with feedback (bottom row). Noise with SD greater than 0.2 drives robust activation of the ‘expected’ phoneme on average, but activations from noise are much lower than activations from intact phonemes. Thus, TISK with feedback generates a plausible pattern of results that are transparently linkable to results with human subjects.

### 2.5. Simulation 5: Graceful degradation

The obvious impact of including feedback in a model is that it can provide a mechanism for simulating (and explaining) top-down effects. A less obvious but crucial consideration is that feedback promotes *graceful degradation*: gradual rather than catastrophic declines in performance given noise or parameter changes (see Magnuson, Mirman, Luthra, Strauss & Harris, 2018, for extended discussion as well as demonstrations that feedback in the TRACE model promotes higher accuracy and faster word recognition given noisy inputs). We tested TISK with and without feedback for graceful degradation with series of full-lexicon simulations (that is, one simulation for every word in the original 211-word TRACE lexicon) while gradually increasing the amount of Gaussian noise added to input patterns. At each of 15 levels of noise (SD 0.01 to 0.15 in steps of 0.01), we conducted 15 full-lexicon runs (with SD > 0, the noise would vary and therefore performance might as well; multiple runs allow us to establish more stable performance estimates).

However, there is no reason to suspect that the default TISK parameters represent the *best possible* performance without feedback; these parameters were originally chosen without any consideration for performance under noise. To ensure we were putting the autonomous (no feedback) and feedback versions of TISK on maximally equal footing, we explored the parameter space more fully both with and without feedback. The details of these parameter space explorations are presented in Appendix 1. These explorations led to the ‘optimized’ parameters with and without feedback presented in Table 2 above.

**Figure 10:**
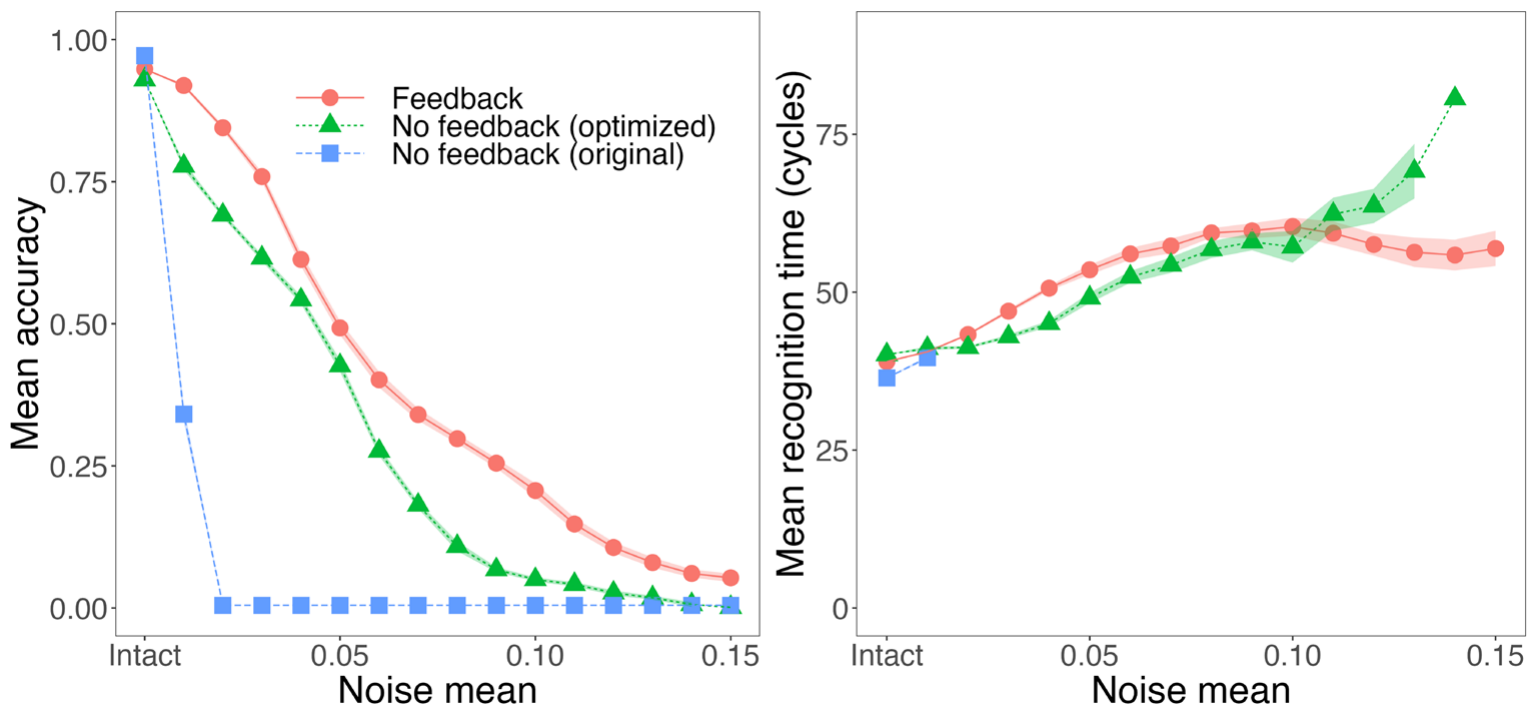
Effects of noise on accuracy and recognition time in TISK with feedback, and three variants of the model without feedback: the original, Hannagan et al. (2013) parameters, the no-feedback parameters optimized for graceful degradation, and the parameters optimized for feedback but with feedback turned off (Simulation 5). Ribbons indicate standard error. Feedback maximizes the ability of the model to exhibit *graceful degradation:* feedback preserves accuracy better under higher levels of noise. In contrast to results with TRACE (Magnuson et al., 2018), the feedback benefit does not extend immediately to recognition time, though an advantage emerges at high levels of noise.

We present results in Figure 10 for accuracy and recognition time. With optimized parameters, TISK exhibits graceful degradation with or without feedback; that is, with a gradual decline in accuracy as noise increases, rather than a collapse (as we see for the original parameters without feedback). However, we do observe a significant advantage from feedback in terms of accuracy.

It is also notable that the optimized feedforward variant of TISK differs markedly from the other models in Figure 6, where we plot model RTs relative to various lexical dimensions. Specifically, it shows weaker associations with Neighbors and Rhymes, and a reversed relationship with ex-embeddings. We have not attempted to determine why this model differs from the others in these ways, as we expect the theoretical gain from such inquiry would be slight at best.

We noted earlier that Magnuson et al. (2018) conducted similar explorations with TRACE. Magnuson et al. (2018) observed catastrophic degradation for TRACE without feedback, and graceful degradation with feedback. They also observed a recognition time advantage for feedback even without noise (see Magnuson et al., 2024, for a replication using raw TRACE activations rather than response probabilities). Curiously, as can be seen in the right panel of Figure 10, recognition times in TISK tend to be *longer* with feedback until we reach the highest levels of noise. What might explain this difference? The most notable difference is that the default parameters for TRACE were optimized for running the model *with* feedback. When Magnuson et al. compared TRACE with and without feedback, it was a matter of *removing* feedback from the feedback-optimized parameters. We took a different tack here, in terms of finding maximally robust parameters without feedback. A question for future research is whether better performance might be possible with TRACE without feedback.

**Figure 11:**
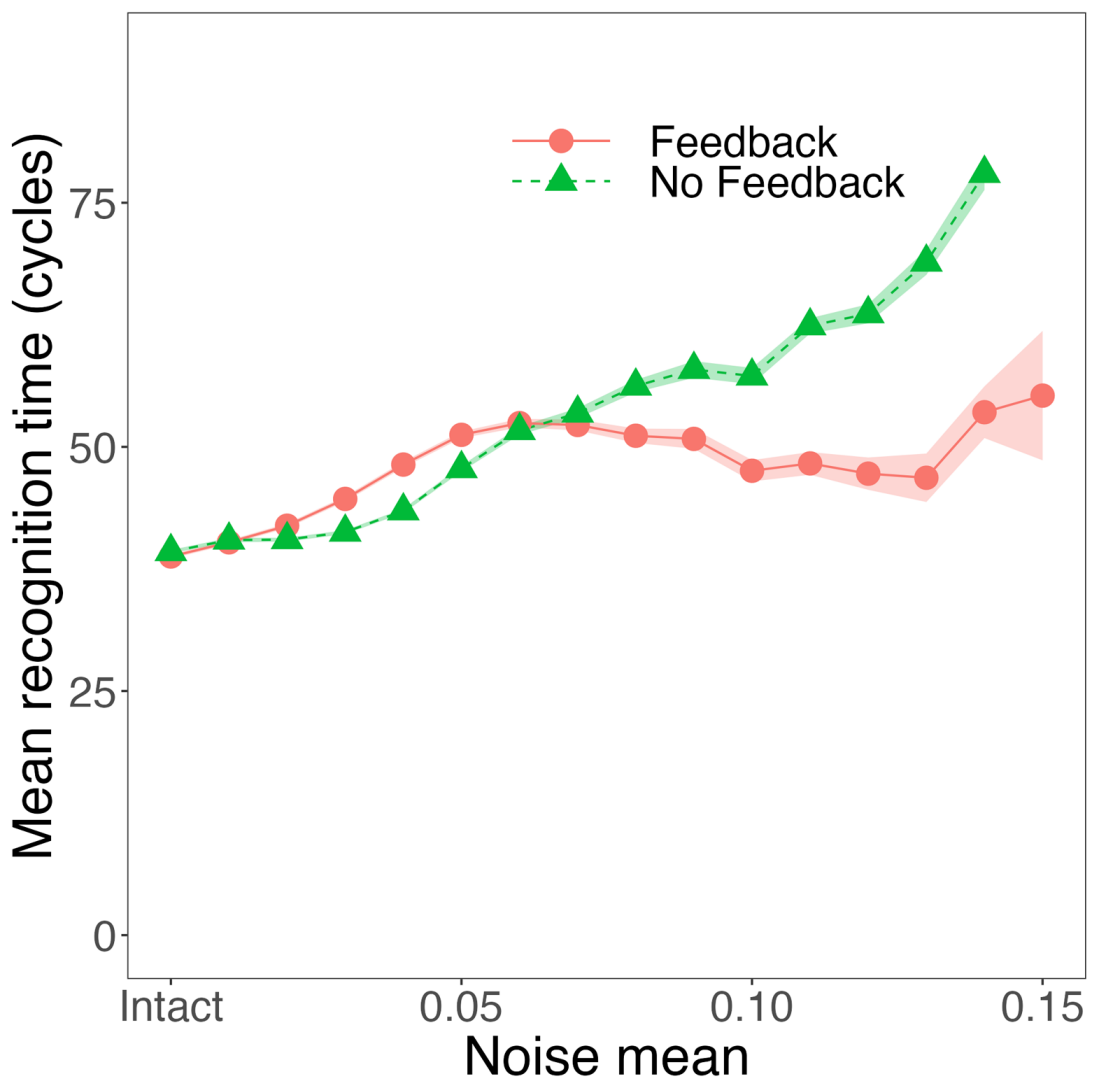
Effects of noise on accuracy and recognition time in TISK with feedback and without (with optimized parameters), but restricted to words that were recognized by both models. This reveals a smaller initial difference and earlier cross-over to a feedback advantage compared to Figure 10. This suggests that the apparent disadvantage for feedback is largely due to the additional words the model with feedback can recognize at higher levels of noise. Ribbons indicate standard error.

However, another possibility is that the RT differences could be related to the accuracy differences. Specifically, the words that the model with feedback is able to recognize but are not recognized by the model without feedback could be particularly challenging items, and that could substantially raise the mean RT for the model with feedback. To investigate this possibility, we restrict the means to only include words that both models (with and without feedback) recognize. The results are showing in Figure 11. Here we see a more modest disadvantage for feedback, and a much earlier switch to a feedback advantage (when noise > 0.6).

**Figure 12:**
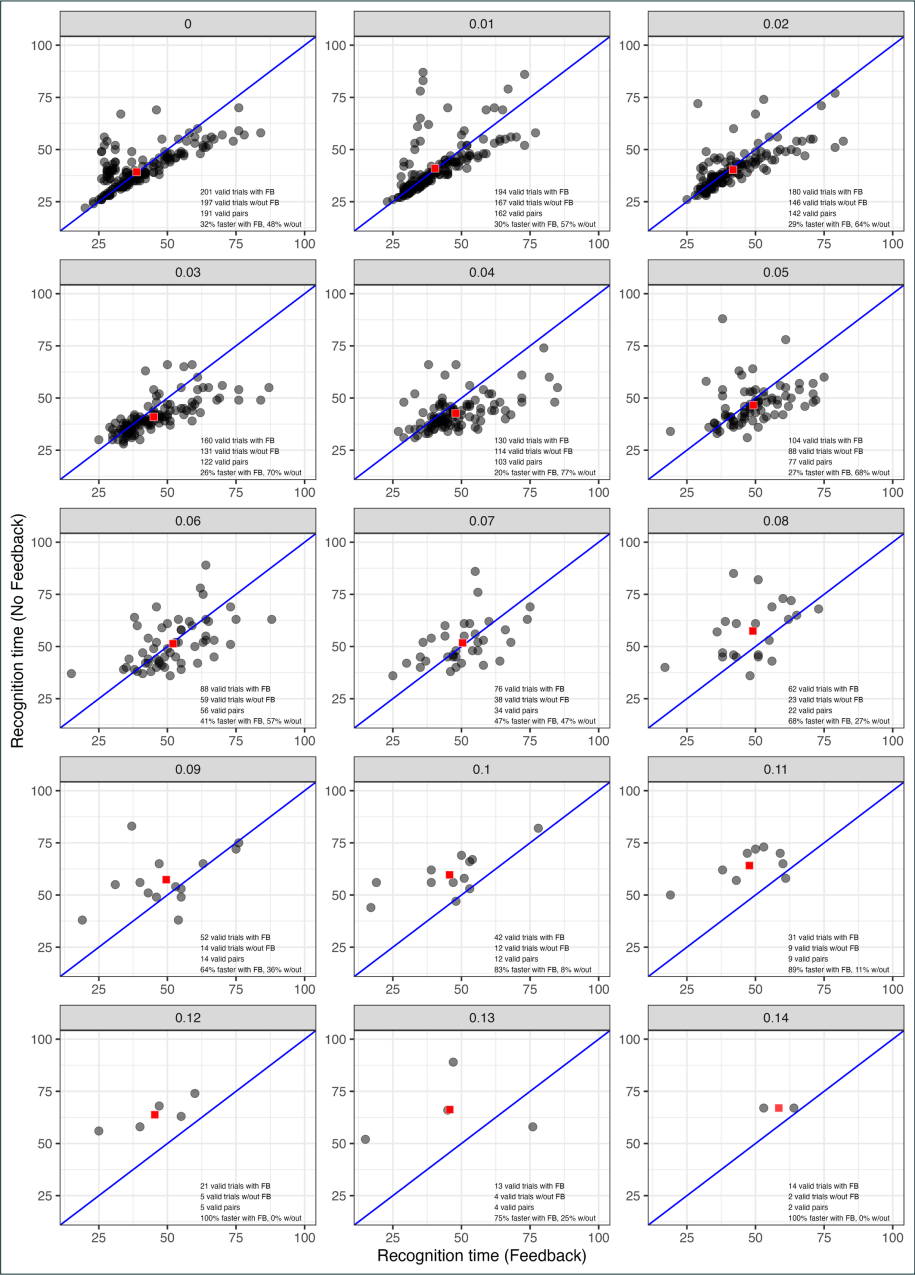
Effects of noise on recognition time in TISK with and without feedback for one model run. Each panel’s label indicates the noise level. Red squares plot mean RT with and without feedback.

**Figure 13:**
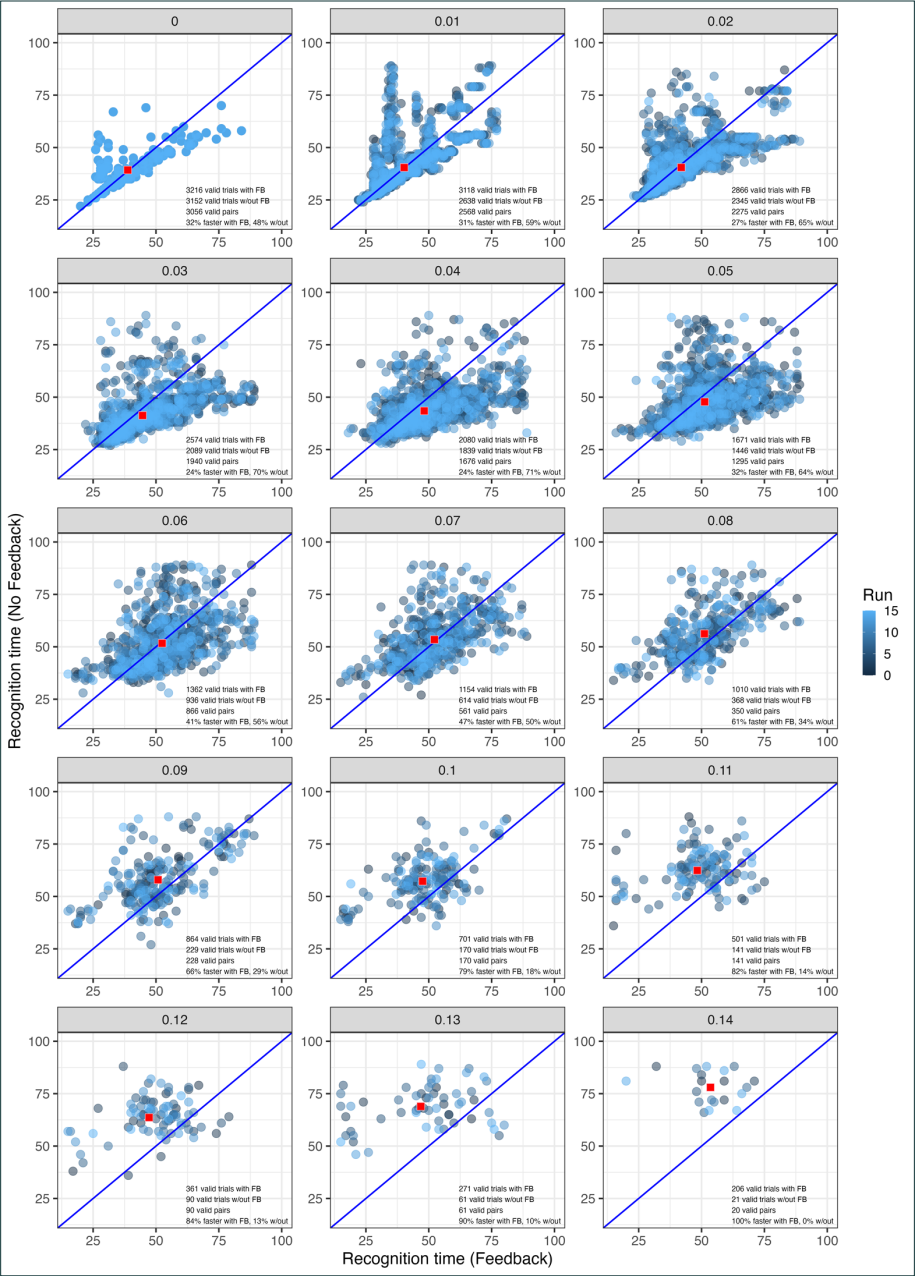
Effects of noise on recognition time in TISK with and without feedback for all 15 model runs. Each panel’s label indicates the noise level. Red squares plot the mean RT values with and without feedback. Color indicates run.

To probe this further, we created scatter plots for one model run (Figure 12) and all 15 runs combined (Figure 13). These plots only include points for words that were recognized by both models at a particular noise level on a specific run. Each panel also includes annotations indicating how many valid cases (i.e., recognized words) there were with and without feedback, how many valid pairs could be plotted (those are words that were recognized by both models at that level of noise), and what proportion of words were recognized more quickly with or without feedback. Red squares in each panel plot mean RT for the two models. This mean value tends to be very close to the identity line even when RT is lower without feedback for a majority of items. Eventually, when noise level reaches 0.07, the mean point rises above the identity line (indicating faster mean RT with feedback than without). Compare this to Figure 10, where the RT lines cross later (at noise = 0.11). Thus, while the impact of feedback on RT is more modest than Magnuson et al. (2018) observed with the TRACE model, it seems that the tendency for higher RTs with feedback in Figure 10 is largely driven by the more challenging words that the model with feedback is able to continue recognizing at higher noise levels.

## 3. Discussion

We set out to examine whether feedback could be added to the TISK model (a) without diminishing its ability to simulate phenomena to which it had already been applied by Hannagan et al. (2013) while (b) providing a basis for plausibly simulating classic top-down effects in spoken word recognition and (c) making the model capable of graceful degradation as inputs become noisy. Our five sets of simulations affirmed that all three of these were the case. Simulation 1 confirmed that with feedback added, TISK remains able to simulate effects to which it had previously been applied (Hannagan et al., 2013); it continues to perform similarly to TRACE (McClelland & Elman, 1986) in terms of the time course of activation of targets and categories of phonological relatives, as well as in terms of item-specific recognition times, and associations of those recognition times with a variety of lexical dimensions (length, numbers of potential competitors, etc.). Simulations 2–4 demonstrated the ability of TISK with feedback to plausibly simulate the Ganong effect, retroactive disambiguation from lexical context, and phoneme restoration, respectively. Finally, Simulation 5 demonstrated graceful degradation: as we added increasing levels of noise to inputs, and compared TISK with and without feedback, we found that (a) TISK exhibits graceful degradation with feedback, (b) without feedback (and with the original TISK parameters from Hannagan et al., 2013), TISK exhibits catastrophic degradation (sudden collapse of accuracy under modest levels of noise), although (c) we were able to find parameter combinations that promote more graceful degradation without feedback, but with a concomitant decline in the model’s ability to exhibit human-like time course of lexical activation and competition.

Simulation 5 is particularly critical with respect to theoretical debates in spoken word recognition. Norris, Cutler and McQueen (2000; 2016) have argued that there is no logical reason to include feedback in models of spoken word recognition. The crucial aspects of their argument are that (a) a system with feedback is more complex than one without, (b) any result that can be simulated with feedback can be simulated in a purely feedforward (“autonomous”) system, and therefore (c) there can be no general benefit of feedback; the best a system can do is tune its feedforward connections to provide the best estimate of the probability of each phoneme given some stretch of input, and appealing to lexical knowledge cannot improve recognition. The details of their argument are considered in depth by Magnuson et al. (2018), who also demonstrate that feedback in TRACE affords graceful degradation even more dramatically than we saw here for TISK.^7^ Magnuson, Crinnion, Luthra, Gaston and Grubb (2024) go further and detail how the joint effects of feedback and lexical activation selectively reinforce lexically-coherent activation patterns over noise. However, Magnuson et al. (2018) did not explore the TRACE parameter space to determine whether parameter combinations are possible that would promote more graceful degradation in TRACE without feedback. This is a possibility that could be pursued in future research, but our primary concern here is the TISK model.

We also note that many findings in spoken word recognition have not yet been tested with TISK. Magnuson and Crinnion (2022) provide a table listing the ~30 distinct results TRACE simulates in spoken word recognition. This provides an obvious agenda for extending TISK in the future.

## 4. Conclusions

Our aim was primarily to gauge TISK’s plausibility by increasing its scope to top-down effects by adding lexical-to-N-phone feedback. TISK already exhibited remarkable similarity to TRACE without feedback (McClelland & Elman, 1986). With feedback, it retains its previous similarity to TRACE while providing plausible simulations of classic top-down phenomena and demonstrating graceful degradation given increasingly noisy inputs (all similar to results previously observed with the TRACE model).

These similarities are all the more remarkable given the architectural differences between TISK and TRACE. To solve the problems of encoding sequence order, including sequences with repeated elements, TRACE employs a “time-as-space” memory with many time-specific copies of each feature, phoneme, and word node. These copies allow TRACE to encode sequences and repeated elements (whether features, phonemes, or words) because each time-specific copy is independent. However, scaling to a realistic size (expanding from 14 phonemes to 40 and from 200 words to 20,000) would require massive numbers of nodes and connections (approximately 1.3 million nodes and more than 40 billion connections). As we discussed earlier (see also Hannagan et al., 2013), we would not argue that these counts by themselves suggest that TRACE’s solution is implausible (e.g., considered in the context of estimates of 86 billion neurons and 150 trillion in the typical adult human brain; Azevedo et al., 2009). However, they raise the question of whether a more computationally economical solution might be possible. TISK (Hannagan et al., 2013) replaces TRACE’s time-specific phoneme and word nodes with time-invariant nodes – that is, single instances. It does this by using not just phonemes at the sublexical level, but also (semi-open) diphones (which is why that layer is called the *N-phone* layer). We describe the diphones as semi-open because, as discussed earlier, time-specific phonemic inputs are mapped to time-invariant diphones in a graded fashion. The /sa/ node would be slightly more activated given *sock* where its constituents are adjacent than in *stock* where there is a one-phoneme gap, which would activate /sa/ more than *strong*, where the gap would be two phonemes. Open diphone counts provide surprisingly distinctive codes; the gradient activation from symmetry coding is even more distinctive, and allows distinctive patterns of activation for ordered sequences and sequences including repeated elements. Feedback in TISK differs from that in TRACE in one other respect: it uses both positive and negative top-down lexical feedback. Positive feedback (to constituents) is much stronger, but we discovered that a small amount of negative feedback (to non-constituents) promoted stable performance.

Again, the similarities in performance despite these differences are remarkable. One might suppose they are attributable to fundamental aspects of the interactive activation architecture used by both TISK and TRACE. However, other models, including simple recurrent networks (Elman, 1990) that are not interactive activation models exhibit remarkable similarity to TISK and TRACE (Magnuson, in preparation). It may be that the information processing constraints of spoken word recognition (mapping sequences “left-to-right” onto forms in lexical memory) are such that any system capable of simulating a few key aspects of the microstructure of human spoken word recognition (e.g., the time course of activation of words overlapping at onset and offset) will necessarily demonstrate similar time course (Figure 4) and associations with lexical dimensions (Figure 5). While we cannot conclude that there are no significant differences between TISK and TRACE, we have not yet discovered any. However, TISK’s successes reported here demonstrate that a key criticism of TRACE – concerning its reduplicated, time-specific nodes – does not apply to all instances of interactive activation models of human spoken word recognition.

## Data Accessibility Statement

All scripts required to replicate simulations, analyses, and graphs are available at https://github.com/maglab-uconn/TISK_FEEDBACK.

## Figures and Tables

**Figure A1:**
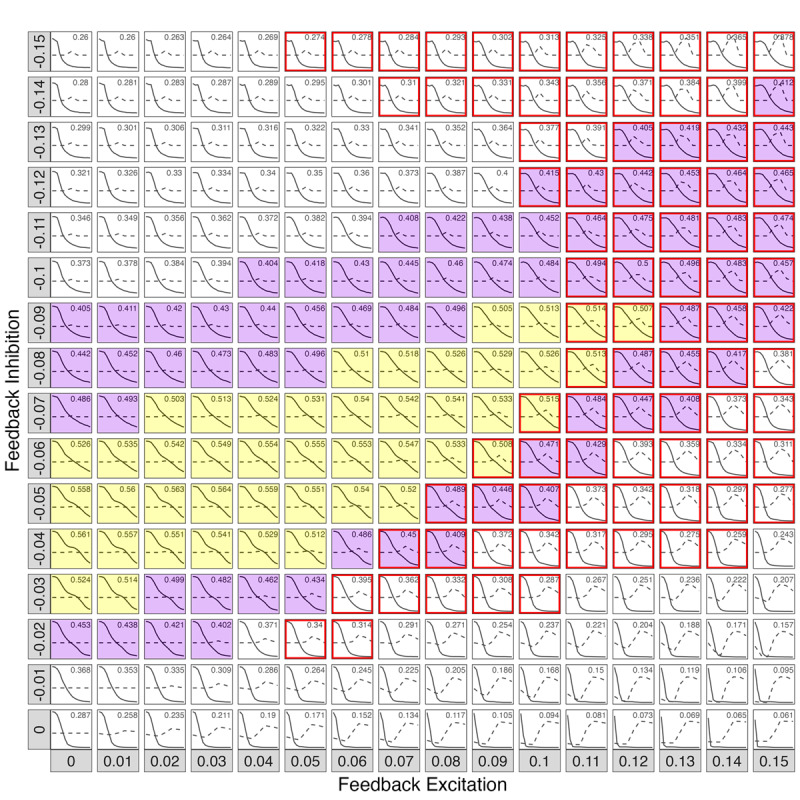
Exploration of positive (x-axis) and negative (y-axis) feedback. In each panel, the solid line is the ‘graceful degradation’ result (see Figure 11) and the dashed line is the Ganong effect. The number in the upper right of each panel is mean accuracy over the full range of noise in the graceful degradation simulations. Panels are shaded yellow if mean accuracy in graceful degradation is > 0.5, or purple if mean accuracy was > 0.4. Panels have red outlines if there is a plausible Ganong effect (maximum difference ≥ 0.15, minimum > 0). Informally, we consider panels that are yellow or purple and highlighted in red to indicate parameter ranges that result in robust performance with feedback (approximately 16% of the combinations explored here).

**Figure A2:**
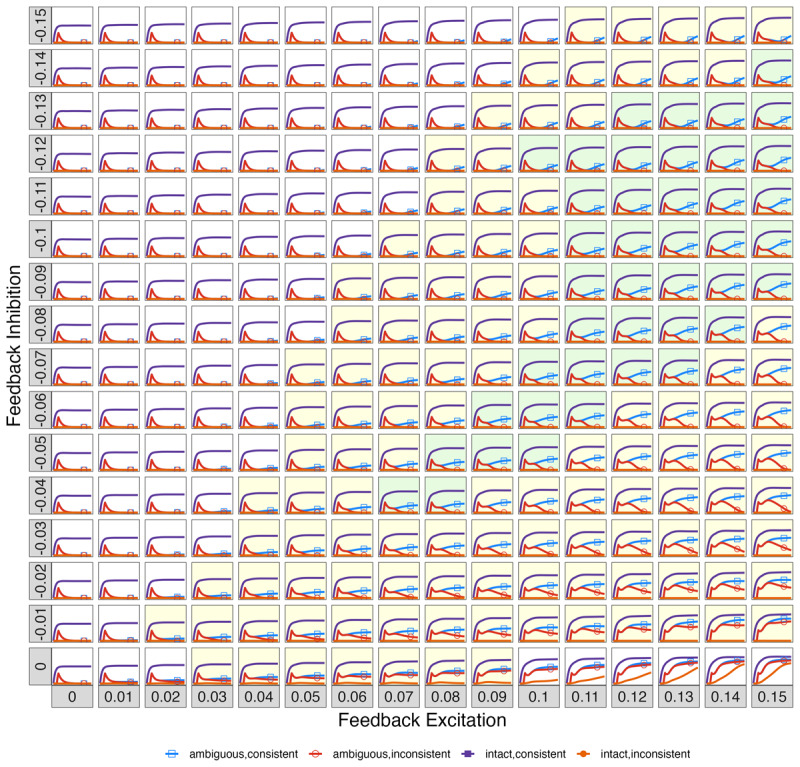
Further exploration of positive (x-axis) and negative (y-axis) feedback. In each panel, retroactive lexical influence simulations (as in Figure 8) are plotted with different feedback parameters. For simplicity, intact or ambiguous cases that are lexically consistent or inconsistent are averaged. Cases where, given ambiguous input, the lexically consistent phoneme’s activation excedes the inconsistent phoneme’s by 0.05 and, given consistent input, the lexically inconsistent phoneme’s activation does not excede 0.05 are shaded yellow or green. Green shading indicates cases that yield robust graceful degredation in Figure A1 (yellow or purple shading with red outline). Thus, a fairly broad range of parameters yields robust performance with feedback (green shading corresponds to ~16% of explored combinations, which includes all cases shaded in yellow or purple and outlined in red in Figure A1).

**Figure A3:**
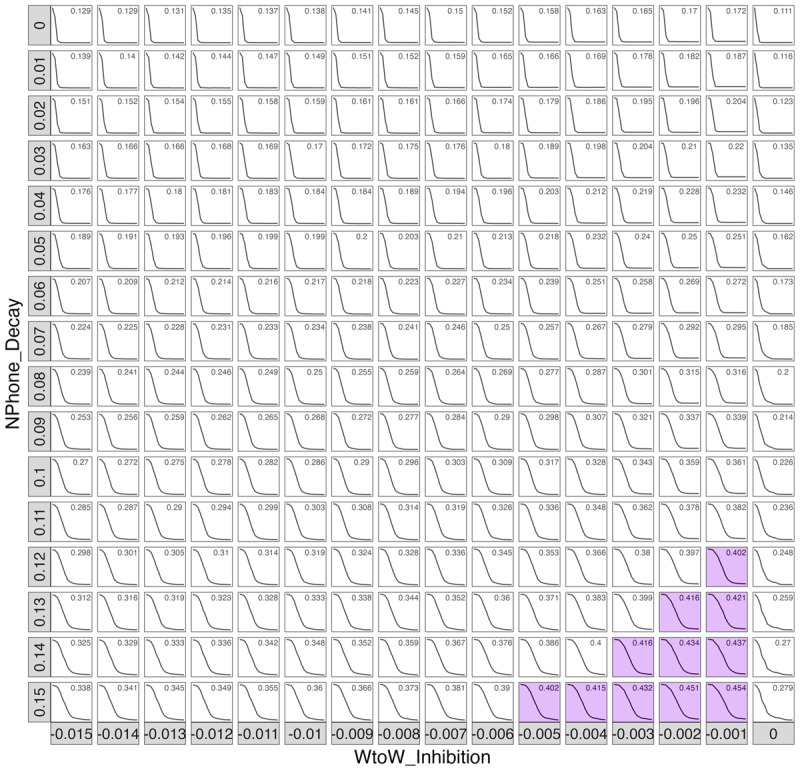
Parameter exploration without feedback. This figure shows graceful degradation results as a function of word-to-word inhibition (x-axis) and N-phone decay (y-axis) with other parameters already optimized. Parameters outside these ranges yield unstable results. A fairly narrow range of parameters (approximately 4% of explored combinations) leads to fairly robust graceful degradation results (purple shading indicates combinations that yield mean accuracy over noise levels > 0.4).

## Footnotes

Shortlist B (Norris & McQueen, 2008) is a provocative outlier both in its rejection of considering ‘activation’ in favor of probability, but also the lack of an explicit competition mechanism; when probabilities are calculated, ‘competition’ is implied from the zero-sum ‘game’ of calculating probabilities.

TRACE only has 14 phonemes; typically, instances of /æ/ are coded as /a/ in TRACE.

If we include a “blank” for the second position, we can also encode each single phoneme in a word, crucially providing a means for including words consisting of a single phoneme.

A kernel function in mathematics translates a mapping (typically, a nonlinear mapping) to a matrix form where the original function can be expressed as a matrix operation (typically inner product). A *string kernel* is a variant of this where the mapping is between strings. For words, one of the key challenges this addresses is differences in word length. If we can recode words as the frequencies of occurrences of distinct diphones (in a phoneme-by-phoneme matrix, or as bigrams in a letter-by-letter matrix for visual words), we translate each word to a same-dimensional object (the element-by-element matrix). Then we can potentially compute things like word-to-word similarity using matrix operations. In TISK, we also leverage this idea to recode the ‘templates’ for words as the set of ordered (open) diphones they contain (open diphones allow gaps between phoneme pairs, such that /kæt/ has the ordered open diphones /kæ/, /kt/, and /æt/). This is what allows TISK to replace the large number of reduplicated, time-specific phoneme and word templates required by TRACE with time-invariant open diphones (as well as phoneme nodes).

The models differ in that 0.0 is the lowest possible activation in TISK while activations can become negative in TRACE; hence, rank order is the crucial concern. Note that negative activations in TRACE can be easily transformed to positive predictions using, e.g., the Luce choice rule (R.D. Luce, 1959; cf. Allopenna, Magnuson, & Tanenhaus, 1998).

Noise with SD > 0.2 drives similar phoneme activations as intact phonemes in TISK without feedback due to the threshold function governing activation. When the noise SD is 0.2 or less, total input to the phoneme node (a combination of bottom-up input and its own previous state adjusted by decay) does not reach threshold and so its resulting activation remains 0. When noise SD > 0.2, the threshold is reached, and the ‘missing’ phoneme still reaches saturation-level activation. With the parameters optimized for feedback, the maximum (saturation) level is approximately 0.62, but noise inputs do not drive phoneme activations near that level, resulting in qualitatively different levels of phoneme activation given silence, noise, or intact input.

For more on this ongoing debate, see Magnuson and Luthra (under review), and for robust and replicated empirical support for interaction (feedback), see Luthra et al. (2021; and for discussion of those results, see McQueen, Jesse & Mitterer [2023] and Luthra et al. [in press]).

## Appendix 1: Parameter space exploration

To optimize parameters with feedback, we explored the space defined by the parameters shown in Table 2. We do not present results from the full exploration, which involved thousands more simulations. In Figure A1, we present results across a range of positive and inhibitory feedback parameters (with other parameters already optimized). Panels highlighted in red with yellow or purple shading indicate combinations that yield robust Ganong effects (cf. Figure 8) as well as robust graceful degradation (Figure 11). In Figure A2, we present retroactive phoneme restoration simulations (cf. Figure 8) as a function of feedback parameters. Yellow shading indicates robust phoneme restoration. Green shading indicate panels that show robust retroactive phoneme restoration as well as robust Ganong effects and graceful degradation (i.e., panels that would have yellow or purple highlighting and a red outline in Figure A1). In Figure A3, we present results from the parameter exploration without feedback. The purple-shaded panels indicate parameter combinations that yield fairly robust graceful degradation results (cf. Figure 11).
